# The Long Term Effect of Current and New Interventions on the New Case Detection of Leprosy: A Modeling Study

**DOI:** 10.1371/journal.pntd.0001330

**Published:** 2011-09-20

**Authors:** Egil A. J. Fischer, Sake J. de Vlas, J. Dik F Habbema, Jan Hendrik Richardus

**Affiliations:** Department of Public Health, Erasmus MC, University Medical Center Rotterdam, Rotterdam, The Netherlands; Kwame Nkrumah University of Science and Technology (KNUST) School of Medical Sciences, Ghana

## Abstract

**Background:**

Although the number of newly detected leprosy cases has decreased globally, a quarter of a million new cases are detected annually and eradication remains far away. Current options for leprosy prevention are contact tracing and BCG vaccination of infants. Future options may include chemoprophylaxis and early diagnosis of subclinical infections. This study compared the predicted trends in leprosy case detection of future intervention strategies.

**Methods:**

Seven leprosy intervention scenarios were investigated with a microsimulation model (SIMCOLEP) to predict future leprosy trends. The baseline scenario consisted of passive case detection, multidrug therapy, contact tracing, and BCG vaccination of infants. The other six scenarios were modifications of the baseline, as follows: no contact tracing; with chemoprophylaxis; with early diagnosis of subclinical infections; replacement of the BCG vaccine with a new tuberculosis vaccine ineffective against *Mycobacterium leprae* (“no BCG”); no BCG with chemoprophylaxis; and no BCG with early diagnosis.

**Findings:**

Without contact tracing, the model predicted an initial drop in the new case detection rate due to a delay in detecting clinical cases among contacts. Eventually, this scenario would lead to new case detection rates higher than the baseline program. Both chemoprophylaxis and early diagnosis would prevent new cases due to a reduction of the infectious period of subclinical cases by detection and cure of these cases. Also, replacing BCG would increase the new case detection rate of leprosy, but this effect could be offset with either chemoprophylaxis or early diagnosis.

**Conclusions:**

This study showed that the leprosy incidence would be reduced substantially by good BCG vaccine coverage and the combined strategies of contact tracing, early diagnosis, and treatment of infection and/or chemoprophylaxis among household contacts. To effectively interrupt the transmission of *M. leprae*, it is crucial to continue developing immuno- and chemoprophylaxis strategies and an effective test for diagnosing subclinical infections.

## Author Summary

Leprosy is a contagious disease that will remain prevalent, despite the declining number of patients worldwide over the last century. With approximately 250,000 new cases detected annually, leprosy is far from being eradicated. Leprosy can be treated with drugs after disease detection.

Some cases can be prevented with a tuberculosis vaccine (BCG) that cross-reacts with the bacterium responsible for leprosy, but this vaccine might be replaced in the future. Furthermore, preventive drugs can reduce the number of new cases among people in contact with infectious patients, but this strategy has not yet become established in common practice. Also, a new test is under development for the detection of infections before the appearance of symptoms.

In this study, we used a computer model to assess the effectiveness of seven possible leprosy control activities. Our results showed that the decline in incidence of leprosy would slow down or halt with the introduction of a new tuberculosis vaccine that is ineffective against leprosy. However, this effect could be offset by the implementation of effective tests for early diagnosis or the routine administration of preventative drugs to contacts of patients.

## Introduction

The global new case detection rate of leprosy has dropped considerably during last century, but with approximately 250,000 new cases detected annually, leprosy is far from being eradicated [1]. Currently, the primary strategy for controlling leprosy is case detection and treatment with multidrug therapy (MDT). Although new interventions are under development, their potential impact on disease control is unknown. Recent clinical trials have indicated that a single chemoprophylactic dose of rifampicin given to individuals in contact with newly diagnosed leprosy patients could protect these contacts against leprosy disease [2]. The results with a single dose of rifampicin are very comparable to trials with dapsone that were conducted in the pre-MDT era. A meta-analysis showed that the combined results from the randomized controlled trials favored chemoprophylaxis to placebo with 2–4 years of follow-up (relative risk 0.59, 95% (CI) 0.50–0.70) [3]. The advantage of a single dose of rifampicin is that it is only given once, while dapsone prophylaxis is given for at least 2 years. Furthermore, new tests are under development for identifying subclinical infections [4].

Other recent developments however, are cause for concern. For example, the integration of leprosy control activities into general health care programs has in many countries led to the cessation of active case finding and contact tracing. Consequently, diagnosis is delayed and patients are therefore infectious for a longer period causing more people in contact with patients to become infected.

Another concern is that a new vaccine may replace the current Bacillus Calmette-Guérin (BCG) tuberculosis vaccine, which is given to infants to prevent tuberculosis (TB), but which also protects against leprosy [5]. An update on progress describing new TB vaccine candidates that are currently entering clinical trials has recently been published [6]. Most are pre-exposure vaccines and will most likely prevent TB disease. Such vaccines are intended either to replace BCG (recombinant live vaccines) or to be given after BCG prime as boosters (protein adjuvant formulations or recombinant viral carriers). New and more specific TB vaccines may not induce cross-immunity to the bacterium responsible for leprosy, *Mycobacterium leprae* [6], [7]. It was recently pleaded that new candidate vaccines must be developed taking both diseases into account, and that the current TB candidate vaccines should be assessed for their potential to protect against leprosy as well as against TB [8]. Therefore, the effect of new leprosy interventions strategies should be tested in the context of other related developments, such as possible changes to BCG.

Although the short-term effectiveness of new interventions can be assessed in trials, extrapolation to long-term effectiveness in the general population is difficult, due to the complex impact on transmission dynamics. Hence, dynamic simulation models are necessary to assess the possible impact of different intervention strategies on future trends in the new case detection rate of leprosy.

We have developed a microsimulation model that simulates the transmission and control of leprosy (the SIMCOLEP model), taking into account the population structure of households [9]. The model has been quantified by data from northwest Bangladesh in 2003. Very detailed data were available for that year from a large randomised controlled trial of chemoprophylaxis with single dose rifampicin (the COLEP study) that was being conducted at the time [2], [10]–[13]. This is an area with a well-organized leprosy control program and with a decreasing trend in new case detection since the mid-1990′s. Regardless, the current case detection rate remains one of the highest in Bangladesh, 2–3 per 10,000 population. We applied our model to the situation in this area as a starting point for exploring the potential impact of seven different intervention strategies on the detection of new cases of leprosy over a 50-year period.

## Methods

### Ethics statement

For the COLEP trial (ISRCTN 61223447), on which the data of this modeling study is largely based, ethical clearance was obtained from the Ethical Review Committee of the Bangladesh Medical Research Council in Dhaka (ref. no. BMRC/ERC/2001–2004/799). All subjects were informed verbally in their own language and invited to participate. Written consent was requested from each adult. For children consent from a parent or guardian was given.

### The model

The microsimulation model simulates the life history of fictitious individuals [9]. These individuals are members of a household that is formed, changes, and dissolves during the simulation. Individual household movement occurs during adolescence and after marriage. Some married couples start living in the household of the parents-in-law, and will form their own separate household after on average 12 years. The life span of individuals is drawn from a life-table at birth; the number of newborn individuals maintains the simulated population growth rate equal to the observed population growth rate; newly born individuals are placed into the household of their mothers; and mothers are drawn from the population of married women and weighted with an age-dependent fertility function.

An individual that is susceptible to leprosy is defined as an individual that developed leprosy sometime during their lifetime, after acquiring the infection. The large majority (say 80–95%) of the population is assumed not to be susceptible to leprosy [14]–[16].The remaining 5–20% of the population is susceptible. For these individuals, it is assumed that 80% undergoes a self-healing infection and is never infectious to other individuals, that is 20% will become chronically infected and infectious [16].

The mechanisms underlying leprosy susceptibility are currently unknown [9]. Therefore, the model used six hypothetical mechanisms: *Random* (no mechanism, but each individual has a fixed probability of being susceptible); *Household* susceptibility (all susceptibles live in a fraction of households, within these susceptible households a fraction of inhabitants is susceptible); *Dominant* (susceptibility is inherited by a dominant gene); *Recessive* (susceptibility is inherited by a recessive gene); *Household* & *dominant* (50% of susceptibility is determined by the *Household* and 50% by a *dominant* gene); *Household* & *recessive* inheritance (50% of susceptibility is determined by the *Household* and 50% by a *recessive*).

As described in a previous paper [9], the model was unable to identify one single mechanism that could best explain the observed data. However, for *Random* it turned out that 20% susceptibles provided the best fit, whereas this was 10% for the other mechanisms. For *Household* this 10% was established by assuming on average 25% of the households contain on average 40% susceptible individuals.

The quantification of the model is based on the leprosy situation in 2003 and the control program of the last decades in the Nilphamari and Rangpur districts of Bangladesh [9]. This control program consisted of passive case detection, with in 2003 an average detection delay of 2 years, treatment with MDT, and active tracing of people in contact with patients. Contacts are examined annually for three consecutive years. In this area, BCG vaccination was routinely given to newborn infants. Since the introduction of the BCG vaccination in 1974, the coverage had gradually expanded to 80% in 1990 and remained at that level in 2003 [17]. BCG had a protective effect of 60% [18].

For a full and detailed description of the model, we refer to our previous paper [9].

### Intervention strategies

In the study we considered seven potential intervention scenarios for the future control of leprosy. The baseline scenario was the current leprosy control program in the Bangladesh study area, as described above. The other scenarios were modifications of the baseline control program. These other six scenarios were: 1) no contact tracing; 2) with a single chemoprophylactic dose of rifampicin, which cured 50% of subclinical cases, for each individual in contact with a leprosy patient [2]; 3) with diagnosis of subclinical cases with a sensitivity of 70% [19] followed by effective treatment; 4) with all newly born infants in the population receiving a new (hypothetical) tuberculosis vaccine that is ineffective against leprosy instead of BCG (no BCG); 5) with the combination of no BCG and chemoprophylaxis; and 6) with the combination of no BCG and early diagnosis with effective treatment.

In our intervention scenarios, contact tracing, chemoprophylactic treatment and early diagnosis were performed only on household members. Contact tracing was repeated three times in three consecutive years with a 10% probability of loss to follow-up and a 90% of symptomatic cases being detected. Early diagnosis was performed in the same schedule as the contact tracing with three consecutive visits to the household. Chemoprophylactic treatment was given only once after examination, in which 90% of symptomatic cases will be detected.

The simulation of interventions was started based on the quantification of 2003, because a detailed data set [16] was available from the COLEP study conducted during that period. The Bangladesh districts at the time when the COLEP study took place can be seen as fairly representative for other areas in the Indian subcontinent with regard to demography, socio-economic condition, cultural tradition and the organization of the health system, including the leprosy control program. The prevalence rate of leprosy at the time was well above the WHO elimination target of 1 per 10,000 population, which was also the case in many areas in India around the year 2000.

## Results

**Figure 1:**
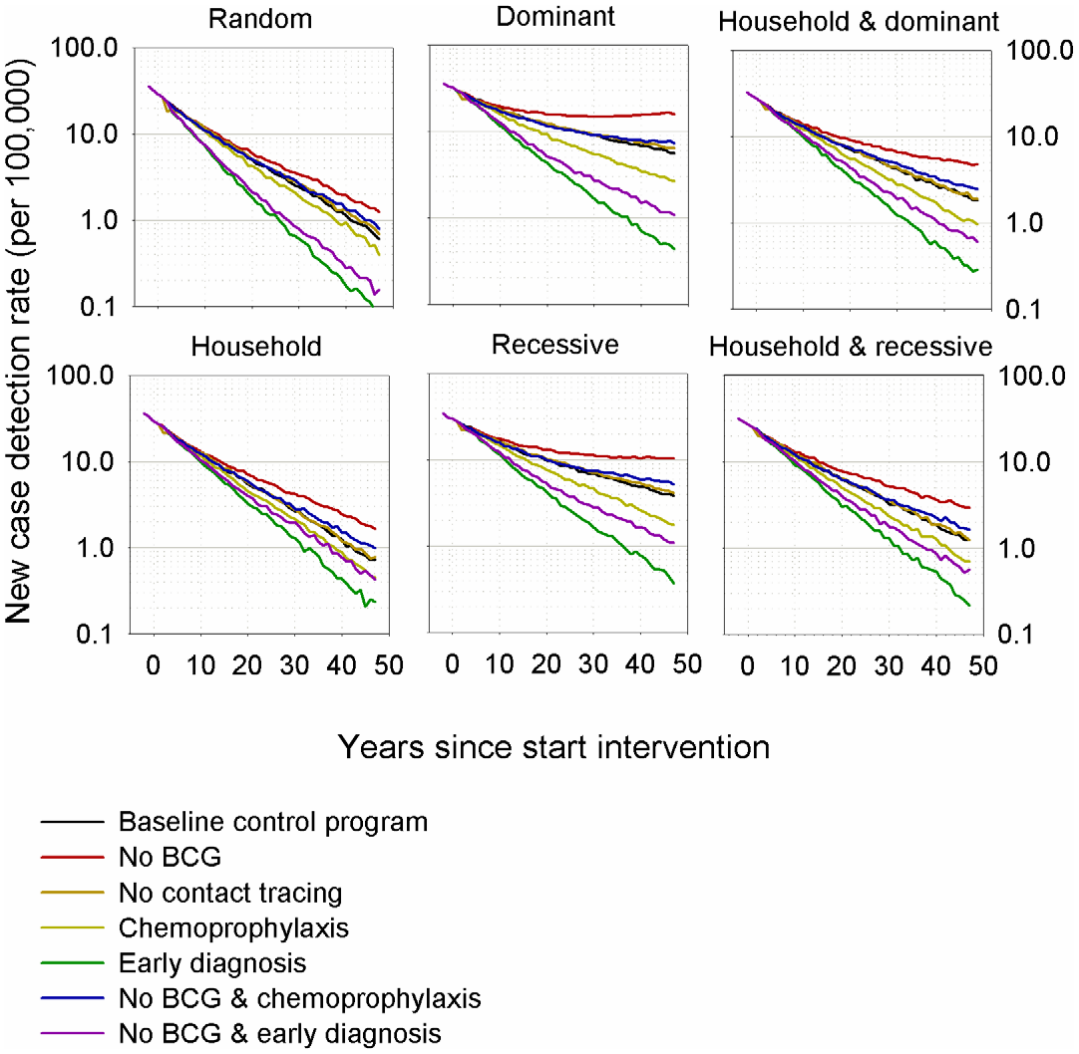
Predicted decline of the new case detection rate with seven intervention scenarios and six mechanisms of leprosy susceptiblity since start of intervention strategy. The baseline program (black line) included passive detection, multidrug therapy, contact tracing, and an infant leprosy-preventative BCG vaccination given at the population level. The other six intervention strategies included the baseline program and, introduction of a new tuberculosis vaccine ineffective against leprosy replacing BCG (red); no tracing of household contacts (orange); a single chemoprophylactic dose of rifampicin that cured 50% of subclinically infected contacts (yellow); early diagnosis of 70% of subclinically infected contacts in each of 3 consecutive annual examinations (green); chemoprophylaxis plus introduction of a tuberculosis vaccine ineffective against leprosy (blue); and detection of subclinically infected contacts plus introduction of a tuberculosis vaccine ineffective against le­pro­sy (purple). Results are the average of 100 runs of the simulation model for each scenario and susceptibilty mechanism.

Table 1 shows the predicted new case detection rates at 25 years after the initiation of the interventions. Under the baseline control program, the different mechanisms that determined susceptibility showed up to three-fold differences in the predicted number of cases per 100,000 people. In Figure 1, the trends in the new case detection rates over 50 years are shown for all seven interventions. All susceptibility mechanisms give qualitatively comparable trends. When the intervention scenarios were ordered after 50 years by the amount of reduction in new case detection rates, the order was as good as identical for all mechanisms; *i.e.* early diagnosis lowest; then no BCG & early diagnosis; then chemoprophylaxis; then baseline; then no BCG & chemoprophylaxis together with no contact tracing; and finally no BCG had the highest new case detection rate.

**Table 1:** Predicted new case detection rates (per 100,000) at 25 years after the introduction of the indicated intervention scenario for six mechanisms of leprosy susceptibility as described in [9].

Intervention	Mechanism determining susceptibilty					
	*Random*	*Household*	*Dominant*	*Recessive*	*Household & dominant*	*Household & recessive*
Baseline control	3.4	4.0	10.4	8.2	5.6	4.6
No BCG	4.6	5.5	15.0	11.9	7.6	6.4
No contact tracing	3.8	4.1	10.5	8.5	5.5	4.8
Chemoprophylaxis	2.8	3.2	6.9	5.9	4.1	3.4
Early diagnosis	1.1	2.1	2.7	2.6	2.1	2.0
No BCG & chemoprophylaxis	3.6	4.0	10.5	8.5	5.9	5.1
No BCG & early diagnosis	1.2	2.5	3.7	3.7	2.9	2.8

The results are average of 100 runs of the simulation model.

**Figure 2:**
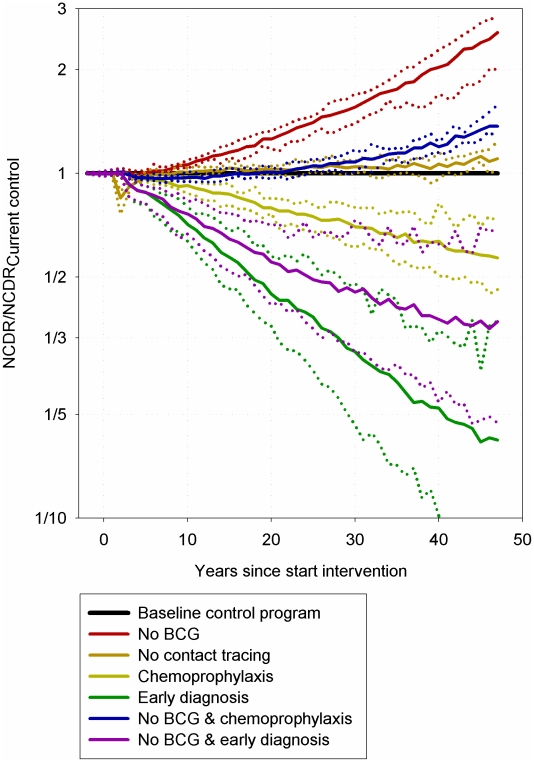
Predicted new case detection rates for six intervention scenarios relative to the baseline control program. For each intervention scenario simulations with different mechanisms of susceptibility to leprosy, as defined in our previous paper [9] are performed. For each intervention scenario, the two dotted lines show the smallest and largest deviations from the baseline control program. The solid line shows the median of all susceptibility mechanisms. Results are the average of 100 runs of the simulation model.

Both the cessation of contact tracing and the replacement of BCG vaccine by a tuberculosis vaccine ineffective for leprosy (no BCG) would have detrimental effects on the rate of decline in leprosy (Figure 2). Twenty-five years after introduction of the ineffective vaccine (no BCG), the new case detection of leprosy was approximately 1.5 times higher than the baseline (Table 1). The cessation of contact tracing was predicted to have a smaller impact, with a marked drop in detection of new leprosy cases during the first few years. This sudden drop was due to the reduced number of examinations of people in contact with patients; thus, these cases would not be detected until later, through passive detection (self-reporting).

Both chemoprophylaxis and early diagnosis were predicted to have substantial effects on the new case detection of leprosy (Figure 2). With no BCG, chemoprophylaxis would partially compensate for the predicted increase in new case detection rates. Furthermore, early diagnosis was predicted to more than compensate for the adverse effects of a leprosy-ineffective tuberculosis vaccine, and reduce the rate of new case detection compared to the baseline. The effects were more promising with the ongoing presence of the BCG vaccine. Under those conditions, at 25 years after the introduction of chemoprophylaxis, the new case detection rate was predicted to be 25% lower than baseline control. Moreover, with the introduction of early diagnosis, the new case detection rate was predicted to halve the baseline incidence after 25 years (Table 1).

**Figure 3:**
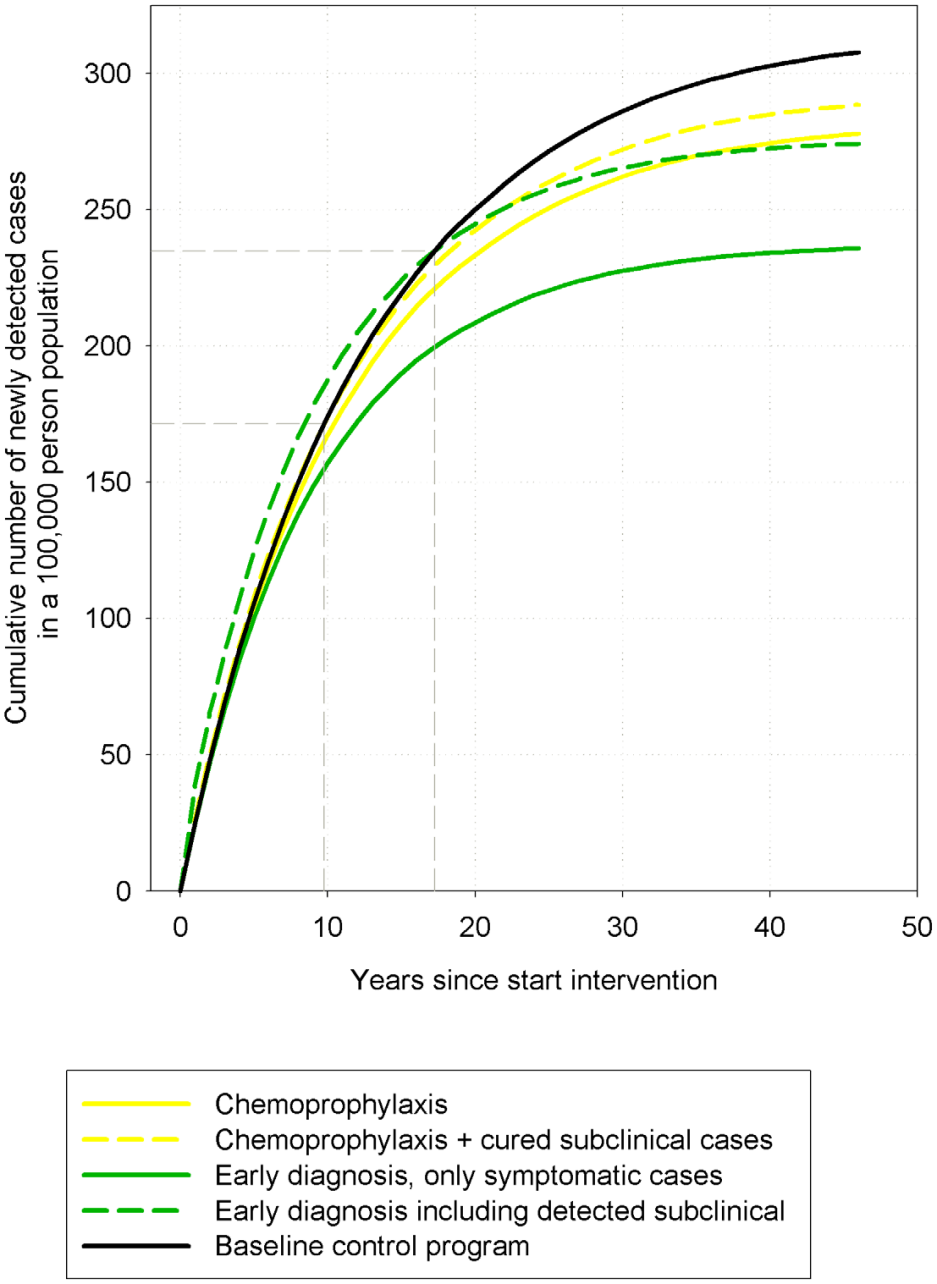
Cumulative New cases detected of leprosy per person-year since start of the interventions. Results are the average of 100 runs of the simulation model.

Early diagnosis of infection allows the detection of subclinical cases, of which part would be detected later or never at all. These subclinical cases are added to the number of detected cases. This is seen in the results of this intervention. The introduction of early diagnosis would increase the total number of detected cases in the first 18 years, simply because of the detection of previously undetectable subclinical cases. Over time however, the total number of new cases (subclinical and clinical) would finally drop below the number detected in the baseline control program (Figure 3). In Figure 3, we show that the new cases detected under the chemoprophylaxis intervention strategy drop immediately below the level of the baseline control program. The additional effect of chemoprophylaxis is that additional new infections are prevented on top of the cure of subclinical infections. These additional prevented infections are due to a shorter infectious period of the cured subclinical infections. To illustrate this effect we show in Figure 3 the clinical and the subclinical cases that were cured by the chemoprophylactic intervention. During the first 10 years, this total number of newly detected cases plus cured cases is equal to the number of newly detected cases under the baseline control program, but afterwards the number of cases plus cured subclinical cases in the chemoprophylaxis intervention group drops under the baseline control program, indicating the prevention of new infections.

## Discussion

This study used a microsimulation model to compare the future outcomes of different leprosy intervention programs. The baseline program consists of passive case detection, treatment with MDT, contact tracing, and infant BCG vaccination. The predicted rate of decline in new case detection depends on the intervention scenario chosen over the next 50 years. Early diagnosis and/or chemoprophylaxis added to the baseline program can result in a considerable reduction in the new case detection rate. Furthermore, these interventions were predicted to compensate for the adverse effect of replacing BCG by a leprosy-ineffective tuberculosis vaccine.

Our microsimulation modeling approach was able to capture individual (stochastic) processes. Complex infection dynamics could thus be simulated on an individual basis. Aggregating the model outcomes enabled the analysis of trends at the population level. The quantification of the model was based on an area, Nilphamari and Rangpur districts in Bangladesh, where leprosy is highly endemic and which has a well-organized control program [10]. The chemoprophylaxis intervention parameters were based on the COLEP trial conducted in this population [10]. Because of the COLEP study, a large amount of very detailed information was available for this population directly prior to the trial that started in 2003 [11]. We did not use data after 2003, because the impact of the chemoprophylactic intervention (a randomised controlled trial) is difficult if not impossible to mimic in our model.

The use of our microsimulation model is limited to interventions on a household basis. A future challenge would be to extend the modeling to interactions between households. This requires data from molecular epidemiology studies, for which techniques only recently have been developed and field tested [20], [21]. Our objective in this study was however to compare interventions that are most feasible, namely targeting household members. The main uncertainty in our modeling is the mechanism that causes susceptibility to leprosy. The transmission parameters as well as future trends differ greatly between these mechanisms [9]. Other parameters will have less influence on the outcomes than these large differences due to these mechanisms. Our conclusions remain the same for all mechanisms.

The absence of data on the households in the years prior to the COLEP trial makes a quantitative validation of the results impossible. Therefore, the conclusions of our study are only qualitative, i.e. ranking of the intervention strategies. However, these can be used to prioritize implementation and fundamental research into chemoprophylactic treatment and early diagnosis. We are confident that our results will apply to Bangladesh and many other regions in the world.

The current leprosy control program in the Nilphamari and Rangpur districts of Bang­la­desh is more extensive than usual. The primary advantage of this program is the active tracing of individuals that had been in contact with newly diagnosed leprosy patients. Contact tracing is not common in leprosy control programs. Our modelling showed that contact tracing and subsequent treatment of newly found patients could, in itself, contribute to a reduction in the transmission of *M. leprae* in the population. Nevertheless, we argue that the qualitative results, *i.e.* the ranking of the intervention strategies, will not differ when implemented in a currrently less intensive control program.

The primary concern of this study was to estimate the relative, not the absolute, impact of the various interventions and take into account alternative hypotheses for mechanisms of susceptibility to leprosy. We compared the results based on different hypothesized mechanisms for susceptibility, because each of these mechanisms could be valid [9]. The quantitative results were sensitive to the mechanism chosen. Nevertheless, when the different interventions were ordered by the magnitude of effect, that order was identical for all the mechanisms of susceptibility. Thus, the qualitative results were robust, and suggested this order of effectiveness for the different interventions can be generalized.

The new interventions chemoprophylaxis and early diagnosis (which necessarily include contact tracing) were predicted to have a clear added impact for leprosy control. We assumed that the effect of chemoprophylaxis with a single dose rifampicin (SDR) could prevent 50% of subclinical infections to develop leprosy. This assumption was based on the outcome of the COLEP trial and represented the overall effect of SDR in the contacts [2]. In the trial, this effect of SDR was a 56% reduction in new leprosy cases after two years for all contacts. The effect of SDR, however, varied among the different types of contacts, with a 49% prevention in neighbors, 54% prevention in household contacts, and 76% prevention in social contacts [2]. Thus, the choice of contacts to be included in contact tracing and subsequent chemoprophylactic treatment is very important. Ideally, it should go beyond the immediate household of the index patient. The choice of the contact ‘ring’ will likely depend on the acceptance of contacts to be involved and the feasibility of running an extended program. Moreover, rather than providing chemoprophylaxis to all, one would prefer to first test for a subclinical infection and then treat individuals appropriately.

Our modeling showed that identification and treatment of subclinical infections among household contacts had the largest effect in reducing transmission of *M. leprae* in the population. Part of the better performance of early diagnosis compared to chemoprophylaxis was that the early diagnosis strategy comprised three consecutive annual tests with 70% sensitivity, compared to a single round of rifampicin with a cure rate of 50%. Thus, more subclinical cases could be cured after the early diagnosis than with chemoprophylaxis. Meima *et al.* [4] showed that a short detection delay is key to the success of the current MDT-based leprosy control strategy. Detection of subclinical cases would be a major improvement because it provides an even shorter detection delay. As shown in Figure 3, the detection of subclinical cases also reduced transmission, and the total number of new cases detected (clinical and subclinical) was predicted to eventually drop below the number of new cases detected under the baseline control program.

Our study shows that BCG may have an important effect on the reduction of the case detection of leprosy. Previously, Meima *et al.* [4] showed, just as in this study, that BCG vaccination may have a large impact on the expected incidence of leprosy in the population. The current knowledge about the effect of the BCG vaccination on leprosy strongly supports maintaining the current BCG vaccination practice [5], [22]. Alternatively, a leprosy-specific compound should be added to an improved tuberculosis vaccine in leprosy endemic areas.

### Conclusions

We showed that the leprosy incidence would be reduced substantially by good BCG vaccine coverage and the combined strategies of contact tracing, early diagnosis, and treatment of infection and/or chemoprophylaxis among household contacts. To effectively interrupt the transmission of *M. leprae*, it is crucial to continue developing immuno- and chemoprophylaxis strategies and an effective test for diagnosing subclinical infections.
